# Chronic Blood Flow Restriction Exercise Improves Objective Physical Function: A Systematic Review

**DOI:** 10.3389/fphys.2019.01058

**Published:** 2019-08-21

**Authors:** Matthew J. Clarkson, Anthony K. May, Stuart A. Warmington

**Affiliations:** School of Exercise and Nutrition Sciences, Institute for Physical Activity and Nutrition, Deakin University, Geelong, VIC, Australia

**Keywords:** blood flow restriction, KAATSU, physical function, exercise, training, activities of daily living

## Abstract

**Background:** Blood flow restriction or KAATSU exercise training is associated with greater muscle mass and strength increases than non-blood flow restriction equivalent exercise. Blood flow restriction exercise has been proposed as a possible alternative to more physically demanding exercise prescriptions (such as high-load/high-intensity resistance training) in a range of clinical and chronic disease populations. While the maintenance of muscle mass and size with reduced musculoskeletal tissue loading appeals in many of these physically impaired populations, there remains a disconnect between some of the desired clinical measures for chronic disease populations and those commonly measured in the literature examining blood flow restriction exercise. While strength does play a vital role in physical function, task-specific objective measures of physical function indicative of activities of daily living are often more clinically relevant and applicable for evaluating the success of medical and surgical interventions or monitoring age- and disease-related physical decline.

**Objective:** To determine whether exercise interventions utilizing blood flow restriction are able to improve objective measures of physical function indicative of activities of daily living.

**Methods:** A systematic search of Medline, Embase, CINAHL, SPORTDiscus, and Springer identified 13 randomized control trials utilizing an exercise intervention combined with blood flow restriction, while measuring at least one objective measure of physical function. Participants were ≥18 years of age. Systematic review of the literature and quality assessment of the included studies used the Cochrane Collaboration's tool for assessing risk bias.

**Results:** Data from 13 studies with a total of 332 participants showed blood flow restriction exercise, regardless of modality, most notably increased performance on the 30 s sit-to-stand and timed up and go tests, and generally improved physical function on other tests including walking tests, variations of sit-to-stand tests, and balance, jumping, and stepping tests.

**Conclusions:** From the evidence available, blood flow restriction exercise of multiple modalities improved objective measures of physical function indicative of activities of daily living.

## Introduction

Strength or resistance training is a primary exercise modality in exercise prescription guidelines for healthy adults (American College of Sports Medicine, 2009), older adults (Nelson et al., 2007), and many clinical populations (Moore et al., 2016). Maintaining or improving muscle mass and strength is imperative for not only higher-level sports performance, but essential musculoskeletal function, which includes common tasks like ambulation, balance, and activities of daily living (ADL) (Garber et al., 2011). Traditionally, maintaining muscle mass and strength with high-load resistance training (HLRT) utilizes loads >70% of an individual's one-repetition maximum (1RM) (American College of Sports Medicine, 2009). However, HLRT is often perceived as being too difficult or technique-intensive for novices (Thiebaud et al., 2014a), or may be contraindicated for certain populations, such as frail individuals, people living with chronic disease, or those in early stage musculoskeletal rehabilitation (Williams et al., 2007; Vanwye et al., 2017). In recent years, low-intensity exercise (20–30% 1RM) combined with blood flow restriction (BFR) has been proposed as a viable alternative to HLRT for maintaining or improving muscle mass and strength (Lixandrao et al., 2018).

A recent systematic review and meta-analysis concluded that HLRT remains a more practical option than low-intensity resistance training with BFR (BFR-RT) for improving strength among individuals able to perform HLRT (Lixandrao et al., 2018). However, development of muscle mass was deemed equally effective with either HLRT or BFR-RT (Lixandrao et al., 2018). Additionally, low-to-moderate intensity aerobic exercise training combined with BFR (BFR-AT) has also been shown to increase muscle mass and strength beyond its non-BFR equivalent (Slysz et al., 2016). While BFR-AT is likely less effective for increasing muscle mass and strength compared with HLRT or BFR-RT, it requires notably less mechanical, haemodynamic and perceptual stress than either HLRT or BFR-RT (May et al., 2017; Neto et al., 2017a; Vanwye et al., 2017). Collectively, BFR-RT and BFR-AT both cater to a broad spectrum of physical abilities among different populations, who may be contraindicated or otherwise opposed to HLRT as a means of maintaining or improving muscle mass and strength.

Both BFR-RT and BFR-AT have been proposed as possible alternatives to more physically demanding exercise prescriptions in a range of clinical and chronic disease populations such as chronic obstructive pulmonary disorder (Thiebaud et al., 2014b), end-stage kidney disease (Clarkson et al., 2017a), ischemic heart disease (Madarame et al., 2013), and inclusion body myositis (Jorgensen et al., 2018). While the ability to maintain muscle mass and size with a reduction in musculoskeletal tissue loading appeals in many of these physically impaired populations, it has not translated into larger scale randomized controlled trials among these populations. Some studies provide proof of concept pilot data among chronic disease populations (McCully et al., 2004; Madarame et al., 2013; Mattar et al., 2014; Jorgensen et al., 2018) or outline the relative haemodynamic safety of the technique (Jessee et al., 2017; May et al., 2017; Neto et al., 2017b; Barili et al., 2018). However, there remains a disconnect between some of the desired clinical measures for chronic disease populations and those commonly measured in the BFR literature. While strength does play a vital role in physical function (Buchner et al., 1997), task-specific objective measures of physical function that are indicative of ADL are often more clinically relevant and applicable for evaluating the success of medical and surgical interventions, or monitoring age- and disease-related physical decline (Groll et al., 2005). As such, the term “physical function” in the present review refers specifically to the ability to independently perform activities of daily living.

Previous systematic reviews of BFR exercise modalities have almost exclusively measured muscle mass and strength with regard to physical outcomes (Loenneke et al., 2012; Slysz et al., 2016; Lixandrao et al., 2018). One review did include mention of the importance of physical function but specifically for musculoskeletal rehabilitation, although this was not a primary focus of the review and instead highlighted the lack of investigation into the examination of physical function with BFR exercise training (Hughes et al., 2017). Other reviews have explored the effects of BFR exercise on bone metabolism (Bittar et al., 2018), haemodynamic responses to BFR exercise (Neto et al., 2017a), or the mechanisms and relative safety of the technique (Fahs et al., 2012; Patterson et al., 2017). Therefore, the purpose of this systematic review was to elucidate the efficacy of both BFR-RT and BFR-AT for improving a range of measures of objective physical function indicative of ADL.

## Methods

### Study Design

This systematic review was conducted in accordance with the Preferred Reporting Items for Systematic Reviews and Meta-Analyses (PRISMA) guidelines.

### Search Strategy

The electronic database search included Medline, Embase, CINAHL, Springer, and SPORTDiscus. Search strategy utilized the search strings identified in the Supplementary Material. Search terms were derived from “physical function,” “blood flow restriction,” and “exercise.” References were also identified in the reference lists of previous systematic reviews in addition to the results of our electronic database search. Search results were filtered within the database where possible for the filters “Human,” “English,” “randomized controlled trial,” “controlled trial,” “clinical trial,” “controlled clinical trial,” “journal,” “journal article,” and/or “academic journal”. Search results included dates from inception until the date of the search (25th November 2018).

### Participants, Interventions, Comparators

**Figure 1:**
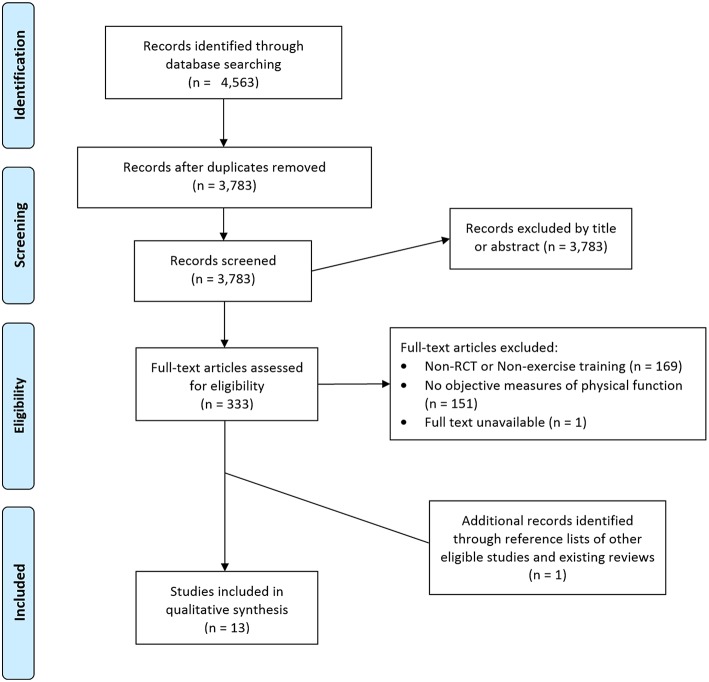
PRISMA flow chart of study selection process.

Database search results were imported into Endnote X8 (Thompson Reuters, Philadelphia, Pennsylvania, USA). Duplicates were removed, and screening was completed by title, abstract, and full text. Excluded articles were sorted into individual folders indicating the reason for exclusion until only articles for inclusion remained. This process was completed by two researchers independently. The relevant inclusion criteria are identified below and reasons for exclusions noted in the PRISMA flow chart (Figure 1):

1. Language: only studies published in English were included in this review.
2. Study Design: only studies that employed a randomized control trial (RCT) design were included. Systematic reviews, narrative reviews, conference abstracts, editorials, letters or publications not-inclusive of original data were excluded.
3. Intervention: studies must have included an exercise training intervention in the form of chronic aerobic, resistance, combined, or alternative types of progressive exercise training or significant chronic muscular activation in the primary intervention group or groups over multiple weeks. The primary intervention group must have used BFR during the prescribed exercise training.
4. Controls: control groups in these studies must have been non-BFR equivalent exercise, non-exercising controls, or alternative traditional exercise prescriptions. Within participant controls (single limb interventions) were excluded from the review.
5. Outcomes: must have included at least one objective measure of physical function indicative of ADL. Subjective measures associated with physical function (questionnaires or surveys) were excluded.

Examples of objective measures of physical function indicative of ADL include the 6-min walk test (6MWT), variations of the sit-to-stand test, balance tests, or grip strength tests, which have similarities in their execution to everyday activities. Measures excluded from this review include laboratory tests such as maximal strength testing, or graded exercise testing utilizing measures of oxygen utilization, ventilatory or lactate threshold, as these are not reflective of ADL.

### Assessment of Risk Bias

The risk of bias of included studies was independently evaluated by two reviewers (MJC, AKM) using the Cochrane Collaboration's tool for assessing risk bias (Higgins and Green, 2011). The overall quality assessment of the RCTs included analysis of both selection bias, detection bias, and attrition bias. Selection bias was examined through method of recruitment, protocol for randomization, concealment of treatment allocation, and similarity of groups' baseline characteristics. Detection bias included blinding of assessors to intervention groups and possible blinding of participants. Attrition bias explored level of adherence of participants, completeness of follow up, and reported reasons for attrition. Contention between quality assessments was resolved through follow up consultation between reviewers. Each component of the bias assessment was assigned a rating of high, low, or unclear risk of bias, sufficient enough to notably impact results or the conclusions of the trial.

### Data Extraction

Following the initial screening, information from identified studies that was extracted included basic study characteristics, mean participant age, sample size, control group intervention modality, and duration, experimental intervention modality and duration, and measures of objective physical function.

## Results

### Literature Search

In total, 4,563 articles were retrieved from searches from inception to 25th November 2018 from Medline (267), Embase (2,154), CINAHL (795), Springer (691), and SPORT Discus (656). Duplicates were removed to refine the total number of articles for screening down to 3,783. Of these 3,783 articles screened for eligibility, 3,450 were excluded based on title or abstract. The full texts of the remaining 333 articles were evaluated based on the inclusion criteria for this review. Of these, 169 were excluded by study design for not being a RCT or not being a training study, 151 were excluded for not measuring an objective measure of physical function indicative of ADL, and 1 full text was unable to be obtained. Ultimately, 12 studies fulfilled the criteria and were included in the current review. An additional article was identified from the reference lists of prior reviews in the field identified as part of the search and was added to the analyses for a total of 13 included studies.

### Study Selection and Characteristics

The studies included in this review are summarized in Table 1 based on sample size, population, exercise modality and duration for both the BFR and control groups, outcome measures, and main findings. The 13 studies included a total of 332 participants. Individual studies generally consisted of small sample sizes, ranging from *n* = 17 (Tennent et al., 2017) to *n* = 48 (Ferraz et al., 2018), with only six studies examining more than the mean number of participants for all studies (26 participants) (Araujo et al., 2015; Bryk et al., 2016; Cook et al., 2017; Barbosa et al., 2018; Ferraz et al., 2018; Ladlow et al., 2018). Among all studies there was a relatively even spread of participants between the BFR intervention groups (mean *n* = 12) and the comparison groups (mean *n* = 12). The populations examined among studies exploring physical function after BFR exercise training were variable. However, the most commonly examined population was older adults (aged 60 years or over) which was examined in six of the included studies (Yokokawa et al., 2008; Abe et al., 2010; Ozaki et al., 2011; Yasuda et al., 2014; Clarkson et al., 2017b; Cook et al., 2017). Other populations examined were generally healthy adults (Ladlow et al., 2018), women with knee osteoarthritis (Bryk et al., 2016; Ferraz et al., 2018), post-menopausal women (Araujo et al., 2015), adults post-arthroscopic knee surgery (Tennent et al., 2017), patients with end-stage kidney disease (Barbosa et al., 2018), and patients with sporadic inclusion body myositis (Jorgensen et al., 2018). The majority of studies employed a control group completing non-BFR equivalent exercise (Ozaki et al., 2011; Araujo et al., 2015; Bryk et al., 2016; Clarkson et al., 2017b; Tennent et al., 2017; Barbosa et al., 2018; Ferraz et al., 2018), or an inactive comparison group (Abe et al., 2010; Yasuda et al., 2014; Cook et al., 2017; Jorgensen et al., 2018). However, three of these studies had a second comparison group; one study utilized an inactive control as well as their non-BFR equivalent exercise group (Araujo et al., 2015), and two studies included a HLRT comparison group in addition to a non-BFR equivalent exercise group (Ferraz et al., 2018), or an inactive control group (Cook et al., 2017). One other study used HLRT as their only comparison group (Ladlow et al., 2018). One study used fundamental balance exercises as an intervention for their comparison group, as this was the common practice exercise prescription for improving the outcome measures assessed in the study, and thus a suitable comparison to the novel use of BFR-RT in their primary intervention group (Yokokawa et al., 2008). Only five studies attempted to report adverse events among participants (Yokokawa et al., 2008; Cook et al., 2017; Tennent et al., 2017; Ferraz et al., 2018; Ladlow et al., 2018). Four of these studies did not have any adverse events (Yokokawa et al., 2008; Cook et al., 2017; Tennent et al., 2017; Ladlow et al., 2018), and one study reported four cases of involved exercise-induced knee pain leading to discontinuation in the study, all of which occurred following HLRT alone (Ferraz et al., 2018).

**Table 1:** Summary of studies evaluating changes in objective measures of physical function following exercise intervention combined with blood flow restriction.

**References**	**Sample (Population, age)**	**Intervention details**				**Comparison group(s)**		**Physical function outcome (BFR vs. comparison)**
		***n***	**Duration**	**Modality**	**BFR prescription**	***n***	**Prescription**	
Jorgensen et al. (2018)	Sporadic inclusion body myositis; 69 ± 6 years	11	12 weeks	Resistance training	10 cm cuff width, 110 mmHg pressure; 2 Sessions per week; 3–4 sets of 25 repetitions of leg press, knee extension, knee flexion, calf raise, dorsi-flexion	11	Inactive control group	↔ 2MWT ↔ 30STS ↔ TUG (no sig. Δ for any measure)
Barbosa et al. (2018)	End-stage kidney disease; ≥ 18 years	12	8 weeks	Resistance training	50% SBP using basic tensiometer; 2 Sessions per week; 6 sets of 10 tennis ball squeezes, 3 sets of 10 bicep curls (1–3 kg), 3 sets of 20 repetitious of 40% 1RM handgrip exercise	14	Non-BFR equivalent exercise group	↔ Handgrip strength (no sig. Δ)
Ladlow et al. (2018)	Healthy Adults; 31 ± 7 years	14	3 weeks	Resistance training	10 cm cuff width, 60% LOP (124 ± 13 mmHg); 9 Sessions per week; 4 sets as 30/15/15/15 repetitions at 30% 1RM for leg press and knee extension	14	HLRT: 4 sets of 6-8 repetitions for deadlift, back squats, lunges at 6RM	↑ Multi-stage locomotion test ↑ Y-Balance test
Ferraz et al. (2018)	Women with knee osteoarthritis; 60 ± 4 years	16	12 weeks	Resistance training	17.5 cm cuff width, 70% LOP (97 ± 8 mmHg); 2 Sessions per week; 4-5 sets of 15 repetitions of leg press and knee extension at 30% 1RM	1. 16. 2. 16.	1. Non-BFR equivalent exercise group 2.HLRT: 4 sets of 10 repetitions of leg press and knee extension at 80% 1RM	↔ 30STS ↔ TUG (no sig. Δ)
Tennent et al. (2017)	Post-operative knee arthroscope; 37 ± 17 years	10	6 weeks	Resistance training	80% LOP; 2 Sessions per week; 4 sets as 30/15/15/15 repetitions of leg press, leg extension, and leg curl at 30% 1RM (*in addition to traditional physical therapy*)	7	Traditional physical therapy for knee arthroscope (immediate weight bearing, immediate formal physical therapy, and unrestricted range of motion)	↔ STS5 ↔ 4SST ↔ Self-selected gait speed ↑ Timed stairs
Clarkson et al. (2017b)	Older adults; 70 ± 7 years	10	6 weeks	Aerobic training	10.5 cm cuff width, 60% LOP (134 ± 4 mmHg); 4 Sessions per week; 10 min walking outdoors at 4 km.h^−1^ (RPE 11-14)	9	Non-BFR equivalent exercise group	↑ 6MWT ↑ 30STS ↑ TUG ↑ QCST
Cook et al. (2017)	Older adults; 76 ± 10 years	12	12 weeks	Resistance training	6 cm cuff width, 150% SBP (184 ± 25 mmHg); 2 Sessions per week; 3 sets to volitional failure of leg extension, leg curl and leg press at 30% 1RM (50% 1RM for leg press)	1. 12 2. 12	1. HLRT: As per BFR protocol, but at 70% 1RM 2.Control group of light mobility exercises with light resistance	↔ Gait speed ↔ SPPB (no sig. Δ for any measure)
Bryk et al. (2016)	Women with knee osteoarthritis; 61 ± 7 years	17	6 weeks	Resistance training	200 mmHg; 3 Sessions per week; 3 sets of 30 repetitions of seated knee extension at 30% 1RM, in addition to non-occluded resistance exercises for hamstring and gluteal muscles at 70% 1RM	17	Non-BFR equivalent exercise group, with knee extensions performed at 70% 1RM	↔ TUG
Araujo et al. (2015)	Post-menopausal women; 54 ± 4 years	10	8 weeks	Hydrotherapy	18 cm cuff width, 80% LOP (106 ± 10 mmHg); 3 Sessions per week; 4 sets as 30/15/15/15 repetitions of hip flexion/extension, hip abduction/adduction, knee flexion/extension (with 1-5 kg ankle weights)	1. 102. 8	1.Non-BFR equivalent exercise group; 2.Inactive control group	↔ STS5 ↔ Gait Speed (no sig. Δ) ↔ Heel-toe walking (no sig. Δ) ↑ TUG
Yasuda et al. (2014)	Older adults; 70 ± 7 years	9	12 weeks	Resistance training	5 cm cuff width, 120-270 mmHg; 2 Sessions per week; 4 sets as 30/25/15/10 repetitions of knee extension and leg press, at 20-30% 1RM	10	Inactive control	↑ 30STS
Ozaki et al. (2011)	Older adults; 66 ± 1 years	10	10 weeks	Aerobic training	140-200 mmHg; 4 Sessions per week; 20 min treadmill walking at 4.5 km.h^−1^ and 1.6 degree incline (45% HRR)	8	Non-BFR equivalent exercise group	↔ 30STS ↑ TUG
Abe et al. (2010)	Older adults; 60 - 78 years	11	6 weeks	Aerobic training	160-200 mmHg; 5 Sessions per week; 20 min treadmill walking at 4 km.h^−1^ (45% HRR)	8	Inactive control	↑ 30STS ↑ TUG
Yokokawa et al. (2008)	Older adults; ≥ 65 years	19	8 weeks	Resistance training	4.5 cm width elastic belt, 120% SBP (70-150 mmHg); 2 Sessions per week; Body weight half squats, forward lunges, calf raises, knee lifts, crunches, seated knee flexion and extension	25	Dynamic balance exercise group performing symmetrical and asymmetrical movements; forward and lateral reach; forward and backward steps; standing and walking on a reduced base of support; increasing the complexity of ambulatory tasks; and functional ankle strengthening, all performed on balance mats.	↔ 10mWT ↔ Jump reaction time ↔ Maximum step distance ↑ TUG

*Age data presented as mean ± SD unless otherwise indicated. BFR, blood flow restriction pressure; SBP, systolic blood pressure; LOP, limb occlusion pressure; HLRT, High intensity resistance training; 1RM, One-repetition maximum (maximum load able to be lifted for a single repetition); 6RM, Six-repetition maximum (maximum load able to be lifted for six repetitions); HRR, Heart rate reserve; RPE, Rating of perceived exertion; 2MWT, Two-minute walk test; 30STS, 30-second sit-to-stand; TUG, Timed up and go; STS5, Five-times sit-to-stand; 4SST, Four square step test; 6MWT, Six-minute walk test; QCST, Queen's college step test; SPPB, Short physical performance battery; 10mWT, Ten meter walk test*.

### Risk of Bias Assessment

#### Selection Bias

One inclusion criteria for this review was that studies had to be randomized controlled trials. Therefore, most studies had adequate randomization or participant allocation (Ozaki et al., 2011; Araujo et al., 2015; Bryk et al., 2016; Clarkson et al., 2017b; Cook et al., 2017; Tennent et al., 2017; Barbosa et al., 2018; Ferraz et al., 2018; Jorgensen et al., 2018; Ladlow et al., 2018). Concealment of the randomization method was adequately described in only five of the included studies (Bryk et al., 2016; Clarkson et al., 2017b; Tennent et al., 2017; Barbosa et al., 2018; Ladlow et al., 2018).

#### Detection Bias

The process used to blind participants and study personnel was adequately described in only a single study, in which the BFR cuffs were also applied to participants in the non-BFR exercise group without inflation as a method of blinding (Barbosa et al., 2018). Collectively, only five studies used blinded assessors for the outcome assessments (Bryk et al., 2016; Tennent et al., 2017; Barbosa et al., 2018; Jorgensen et al., 2018; Ladlow et al., 2018), while one other study displayed a high risk of detection bias due to the outcome assessor being the same researcher who completed all training sessions and statistical analyses in the study (Clarkson et al., 2017b).

#### Attrition Bias

Nine of the thirteen included studies reported attrition and compliance of participants (Yokokawa et al., 2008; Bryk et al., 2016; Clarkson et al., 2017b; Cook et al., 2017; Tennent et al., 2017; Barbosa et al., 2018; Ferraz et al., 2018; Jorgensen et al., 2018; Ladlow et al., 2018). However, one of these only reported the minimum compliance rate for inclusion in the analysis (≥66%) and noted a single participant achieved only 37% compliance (although this was removed in the per protocol analysis) (Jorgensen et al., 2018). In a second study, the BFR exercise group had 5 dropouts (~21%) following initiation of the intervention, compared with 2 from the comparison group (~7%) (Yokokawa et al., 2008). While these dropouts were not included in the baseline analysis, the overall effect sizes may have been affected by the main intervention group having 25% less participants than the comparison group. Eight of the included studies reported either 100% compliance or a specific percentage of the total exercise sessions completed by participants (Yokokawa et al., 2008; Bryk et al., 2016; Clarkson et al., 2017b; Cook et al., 2017; Barbosa et al., 2018; Ferraz et al., 2018; Jorgensen et al., 2018; Ladlow et al., 2018). Compliance ranged from 66% (Jorgensen et al., 2018) to 100% (Bryk et al., 2016; Clarkson et al., 2017b; Ladlow et al., 2018) with a mean compliance rate of 90%. Only three of the included studies identified the intention-to-treat principle when conducting their analyses (Barbosa et al., 2018; Ferraz et al., 2018; Jorgensen et al., 2018).

#### Reporting Bias

There was no clear indication of reporting bias that may limit the interpretation or applicability of the findings from among the included studies. Minor commentary on the reporting of the main outcome data has been included in the limitations section below.

#### Other Sources of Bias

Sample size calculations were only presented in seven of the included studies (Araujo et al., 2015; Bryk et al., 2016; Cook et al., 2017; Barbosa et al., 2018; Ferraz et al., 2018; Jorgensen et al., 2018; Ladlow et al., 2018). Notably, all studies without sample size calculations had a total number of participants that was below the mean number of participants for included studies within this review. This potentially indicates that many of these studies may have been underpowered. One study which did include sample size calculations indicated that they were going to be underpowered before the study even began (63% power at α = 0.05) due to limited access to prospective participants (Jorgensen et al., 2018). Another study used a convenience sample due to time constraints, which is an additional source of bias despite equal randomization within the sample (Ladlow et al., 2018). Other sources of bias included noteworthy acknowledgment of small sample size (Yokokawa et al., 2008; Tennent et al., 2017), and relatively high functioning participants that may have displayed higher physical function than would be reflective of the broader population in question (Yokokawa et al., 2008; Cook et al., 2017).

### Modality and Duration of Interventions

Predominately, exercise training interventions utilized BFR-RT (Yokokawa et al., 2008; Yasuda et al., 2014; Bryk et al., 2016; Cook et al., 2017; Tennent et al., 2017; Barbosa et al., 2018; Ferraz et al., 2018; Jorgensen et al., 2018; Ladlow et al., 2018), although three studies employed BFR-AT (Abe et al., 2010; Ozaki et al., 2011; Clarkson et al., 2017b), and one study utilized BFR during hydrotherapy exercises (similar to BFR-RT) (Araujo et al., 2015). Most interventions ranged from 6 to 12 weeks with only one running for a shorter duration of 3 weeks (Ladlow et al., 2018) and no study durations being longer than 12 weeks. Training sessions occurred twice per week in seven studies (Yokokawa et al., 2008; Yasuda et al., 2014; Cook et al., 2017; Tennent et al., 2017; Barbosa et al., 2018; Ferraz et al., 2018; Jorgensen et al., 2018), three times per week in two studies (Araujo et al., 2015; Bryk et al., 2016), four times per week in two studies (Ozaki et al., 2011; Clarkson et al., 2017b), with participants in one study completing five sessions per week (Abe et al., 2010), and those in another completing 9 sessions per week and resting on weekends (Ladlow et al., 2018). Of the nine studies that provided an indication of session length, sessions ranged from 8 min duration (Ladlow et al., 2018) to 60 min duration (Jorgensen et al., 2018). Loads for the BFR-RT interventions were generally prescribed at 20–30% 1RM for the exercises completed under occlusion, and generally consisted of approximately 75 repetitions (often as 1 set of 30 repetitions followed by 3 sets of 15 repetitions, which is a common prescription among BFR-RT interventions) (Yasuda et al., 2014; Araujo et al., 2015; Bryk et al., 2016; Tennent et al., 2017; Ferraz et al., 2018; Jorgensen et al., 2018; Ladlow et al., 2018). The BFR interventions in the remaining BFR-RT studies utilized training variables closer to traditional HLRT; 3–4 sets of 10–15 repetitions using loads between 40 and 50% 1RM (Yokokawa et al., 2008; Barbosa et al., 2018; Ferraz et al., 2018). Among the three BFR-AT studies included in this review training sessions were 10–20 min in duration and performed at walking speeds of 4–4.5 km.h^−1^ (Abe et al., 2010; Ozaki et al., 2011; Clarkson et al., 2017b), which was noted by the two studies using treadmills as approximately 45% maximal heart rate reserve (Abe et al., 2010; Ozaki et al., 2011), and equivalent to 11–14 on Borg's rating of perceived exertion scale in the outdoor walking study (Clarkson et al., 2017b).

The application of BFR was variable across the included studies. Cuffs were generally pneumatically inflated cuffs capable of regulating pressure, although one study utilized standard tensiometers to apply BFR (Barbosa et al., 2018). Measurements of the cuffs used to apply BFR were detailed in eight of the thirteen included studies, and ranged from 4.5 cm wide (Yokokawa et al., 2008) to 18 cm wide (Araujo et al., 2015). The degree of occlusion and the pressure applied was also variable. Five studies used a relative pressure based on a percentage of total limb occlusion (LOP) (Araujo et al., 2015; Clarkson et al., 2017b; Tennent et al., 2017; Ferraz et al., 2018; Ladlow et al., 2018), or a pseudo-relative pressure based on a percentage of systolic blood pressure (SBP) (Yokokawa et al., 2008; Cook et al., 2017; Barbosa et al., 2018). However, these relative pressures also varied from 60% LOP (Araujo et al., 2015; Clarkson et al., 2017b) to 80% LOP (Tennent et al., 2017), or from 50% SBP (Barbosa et al., 2018) to 150% SBP (Cook et al., 2017). It should be noted that narrower cuffs are generally expected to require greater pressures to achieve the same level of occlusion (Younger et al., 2004; Jessee et al., 2016). However, this relationship is not sufficiently well-characterized, and indeed there is no clear relationship between the degree of LOP and cuff width used among the included studies in this review. Five studies applied an arbitrary pressure range based on pressures used in previous, similar research studies (Abe et al., 2010; Ozaki et al., 2011; Yasuda et al., 2014; Bryk et al., 2016; Jorgensen et al., 2018). Collectively, ten of the thirteen included studies reported the mean pressure or range of progressive pressures used during BFR protocols throughout the study, this ranged from 70 mmHg (Yokokawa et al., 2008) to 270 mmHg (Yasuda et al., 2014).

### Outcome Measures

#### Timed Up and Go

Of the thirteen included studies, eight assessed the timed up and go (TUG) (Yokokawa et al., 2008; Abe et al., 2010; Ozaki et al., 2011; Araujo et al., 2015; Bryk et al., 2016; Clarkson et al., 2017b; Ferraz et al., 2018; Jorgensen et al., 2018). Four of these studies utilized BFR-RT in their interventions (Yokokawa et al., 2008; Bryk et al., 2016; Ferraz et al., 2018; Jorgensen et al., 2018), three utilized BFR-AT (Abe et al., 2010; Ozaki et al., 2011; Clarkson et al., 2017b), and one utilized BFR during hydrotherapy (Araujo et al., 2015). A statistically (*P* < 0.05) and clinically significant decrease in time to complete the TUG was observed in five of these studies and was also a significantly greater decrease in time to complete the TUG (10–15%) than their respective comparison groups (3–6%) (Yokokawa et al., 2008; Abe et al., 2010; Ozaki et al., 2011; Araujo et al., 2015; Clarkson et al., 2017b). Notably, all three of the BFR-AT studies in this review were among these. Two other studies assessing the TUG also saw a significant time effect, however BFR-RT groups were not significantly different to their non-BFR equivalent exercise comparison groups (Bryk et al., 2016; Ferraz et al., 2018).

#### Sit-to-Stand Tests

The most commonly assessed variation of a sit-to-stand test, the 30 s sit-to-stand test (30STS), was assessed by six of the included studies (Abe et al., 2010; Ozaki et al., 2011; Yasuda et al., 2014; Clarkson et al., 2017b; Ferraz et al., 2018; Jorgensen et al., 2018). Three of these studies utilized BFR-RT as an intervention (Abe et al., 2010; Ozaki et al., 2011; Yasuda et al., 2014; Clarkson et al., 2017b; Ferraz et al., 2018; Jorgensen et al., 2018) and three utilized BFR-AT (Abe et al., 2010; Ozaki et al., 2011; Clarkson et al., 2017b). A statistically significant increase in the number of repetitions completed during the 30STS (14–28%), beyond that of the relevant comparison groups (−2–8%), was observed in three of the six studies (Abe et al., 2010; Yasuda et al., 2014; Clarkson et al., 2017b). One other study demonstrated a main effect for time for both the BFR-AT (increasing repetitions by 4 ± 5) and non-BFR equivalent exercise comparison group (increasing repetitions by 2 ± 6), but no statistically significant difference between groups (Ozaki et al., 2011).

The five-time sit-to-stand test (STS5) was assessed in a further two of the included studies (Araujo et al., 2015; Tennent et al., 2017). Both studies reported a time effect, whereby time to complete the STS5 decreased regardless of the type of intervention (−27 to −29%) (Araujo et al., 2015; Tennent et al., 2017). However, neither study demonstrated a group by time interaction or a statistically significant difference between groups (Araujo et al., 2015; Tennent et al., 2017).

#### Gait Speed

Maximal gait speed was assessed in three of the included studies (Yokokawa et al., 2008; Araujo et al., 2015; Cook et al., 2017). Two of these studies utilized BFR-RT as their primary intervention (Yokokawa et al., 2008; Cook et al., 2017), while the other utilized BFR during hydrotherapy (Araujo et al., 2015). None of these studies demonstrated a difference between groups for gait speed. However, one study did demonstrate an overall main effect for time across both intervention groups (Yokokawa et al., 2008). Similarly, one study assessed the change in participant-selected gait speed following a BFR-RT intervention (Tennent et al., 2017). There was a statistically significant time effect (32–37% increase in self-selected gait speed) but no difference between groups (Tennent et al., 2017).

#### Walking Tests

While there was no single walking test assessed in multiple studies, each of the 6-min walk test (6MWT) (Clarkson et al., 2017b), 2-min walk test (2MWT) (Jorgensen et al., 2018), and the multi-stage locomotion test for endurance (MSLT) (Ladlow et al., 2018) were assessed by individual studies. Following a BFR-AT intervention, the distance covered during the 6MWT improved by a significantly greater amount than the improvement seen following non-BFR walking (9 ± 4% vs. 2 ± 1%; mean ± SD) (Clarkson et al., 2017b). In contrast, there were no differences between BFR-RT and inactive controls for distance covered during the 2MWT, and neither group improved over time (Jorgensen et al., 2018). Finally, healthy adults appeared to significantly improve their performance on the MSLT, and thus their endurance, following a BFR-RT intervention by 29% (increasing by 306 ± 246 m, *P* = 0.01), while there was no improvement from baseline following a HLRT intervention (91 ± 341 m, *P* > 0.05) (Ladlow et al., 2018). However, despite the magnitude of the change in MSLT, the authors of this study indicated that there was no statistical difference between the percentage change observed following BFR-RT compared with HLRT (Ladlow et al., 2018).

#### Other Measures of Physical Function

A number of additional objective measures of physical function were assessed in only single studies. These included the Four-square step test (4SST) (Tennent et al., 2017), heel-toe walking for balance (Araujo et al., 2015), jump reaction time (following visual stimuli) (Yokokawa et al., 2008), maximum single step distance (Yokokawa et al., 2008), the Queen's College step test (QCST) (Clarkson et al., 2017b), short physical performance battery (SPPB) (Cook et al., 2017), a timed stair ascent (Tennent et al., 2017), handgrip strength (Barbosa et al., 2018) and the Y-balance test (Ladlow et al., 2018). None of the measures of handgrip strength, heel-toe walking for balance or the SPPB showed any significant improvement or difference between groups following BFR-RT or BFR during hydrotherapy (Araujo et al., 2015; Cook et al., 2017; Barbosa et al., 2018). Each of the 4SST, jump reaction time, and maximum step distance were assessed following BFR-RT interventions, and while each of these studies found a main effect for time across all intervention groups, there was no group by time interactions or any statistically significant difference between groups (Yokokawa et al., 2008; Tennent et al., 2017). Of the remaining measures of physical function, the QCST was assessed before and after a BFR-AT intervention (Clarkson et al., 2017b), while both the timed stair ascent and Y-balance test were assessed before and after BFR-RT interventions (Tennent et al., 2017; Ladlow et al., 2018). Both the QCST and timed stair ascent were associated with a main effect for time across both the BFR and non-BFR equivalent exercise interventions in their respective studies (Clarkson et al., 2017b; Tennent et al., 2017), while the study assessing the Y-balance test found no statistically significant change from baseline for their HLRT comparison group (Ladlow et al., 2018). However, all three groups also found a significant group by time interaction, whereby the BFR interventions significantly improved performance on the QCST, timed stair ascent and Y-balance test beyond their respective comparison groups (Clarkson et al., 2017b; Tennent et al., 2017; Ladlow et al., 2018).

## Discussion

This systematic review provides evidence supporting BFR exercise as a possible alternative for increasing physical function indicative of ADL. This may be especially important for clinical groups and chronic disease populations for which physical function is a key evaluation of the success of medical and surgical interventions, or valuable in monitoring age- and disease-related physical decline (Groll et al., 2005). The benefit would be especially relevant if the populations in question are contraindicated to the mechanical, and perceptual stress associated with HLRT (Vanwye et al., 2017). Indeed, most of the included studies in this review examine populations that may be contraindicated to HLRT. The majority of included studies examined older adults (and one examining post-menopausal women) at greater risk of falls and with a higher incidence of frailty (Frost et al., 2017), and only one examined otherwise healthy adults. The remaining included studies examined chronic disease populations including end-stage kidney disease and sporadic inclusion body myositis or those in need of musculoskeletal rehabilitation or reconditioning following arthroscopic knee surgery, or with knee osteoarthritis. All of these populations encapsulate individuals with functional deficits for whom “traditional” exercise prescriptions may be too challenging or outright contraindicated, and for whom physical function is a valuable surrogate outcome for the success of interventions compared with physical performance outcomes such as absolute strength or maximal cardiovascular fitness.

An important factor to consider when interpreting the findings of this review is the sample sizes employed. Among these, one study identified it was only 63% powered due to recruitment limitations (Jorgensen et al., 2018) and while several studies did not provide sample size calculations, two noted that their small sample size may have been a limitation (Yokokawa et al., 2008; Tennent et al., 2017). Suitable power calculations for the outcome measures employed are necessary to ensure the rigor of future research examining measures of physical function. In order to suitably detect small effect sizes the sample size required is markedly larger than that required to detect large effect sizes. For example, if a study comparing the means of two groups is to be 80% powered with an alpha of 0.05, in order to detect a group by time interaction with an effect size of 0.8 requires 52 participants in total (26 per group), but to detect a smaller effect size of 0.3, 352 total participants are required (176 per group). As such, future research must be more conservative in their sample size estimates and target greater recruitment in order to add weight to the discussion of small effect sizes. However, the findings of those studies with small sample sizes in the present review should not be altogether discounted, but instead be more broadly interpreted as a depiction of the potential application of BFR exercise. The majority (nine) of the included studies examined BFR-RT, which is the most widely employed use of BFR (Slysz et al., 2016; Lixandrao et al., 2018), and one examined BFR during hydrotherapy, which is similar to resistance training performed in the water. Across all studies examining BFR-RT the majority reported either a main effect for time across all groups or a group by time interaction, indicating that BFR-RT may be a suitable alternative to other traditional interventions or non-BFR equivalent exercise training for improving physical function. Interestingly, of the studies that were comparing BFR-RT to inactive controls, two found no significant difference between BFR-RT and the control in physical function, but were among those that noted limitations such as being underpowered or having participants that were higher functioning than the majority of the population they were examining, adding weight to the positive findings among other studies supporting BFR exercise as a possible alternative (Cook et al., 2017; Jorgensen et al., 2018). These accounted for more than half the instances in the present review where the BFR intervention was not effective in improving physical function. Perhaps more importantly, due to the physical limitations among populations for which objective physical function is such an important outcome, almost all measures of physical function following BFR-AT improved to a greater extent than the comparison group. The only measure that did not display this group by time interaction in favor of BFR-AT, still showed a time effect, whereby BFR-AT was equally as effective as the non-BFR equivalent intervention for improving 30STS performance (Ozaki et al., 2011). Given the reduced mechanical, haemodynamic and perceptual stress compared with both HLRT or BFR-RT, this suggests that BFR-AT represents a lot of value as an intervention for populations with pronounced physical impairments (May et al., 2017; Neto et al., 2017a; Vanwye et al., 2017).

The two most prominently used measures of physical function among the included studies in this review were the 30STS (Abe et al., 2010; Ozaki et al., 2011; Yasuda et al., 2014; Clarkson et al., 2017b; Ferraz et al., 2018; Jorgensen et al., 2018) and the TUG (Yokokawa et al., 2008; Abe et al., 2010; Ozaki et al., 2011; Araujo et al., 2015; Bryk et al., 2016; Clarkson et al., 2017b; Ferraz et al., 2018; Jorgensen et al., 2018). As such, the collective results from these measures provides the most information regarding the efficacy of BFR exercise for improving physical function in the present review. Performance on the 30STS, considered an indication of functional lower body strength (Jones et al., 1999), improved following BFR exercise interventions in four of the six included studies (by between 14 and 28%) (Abe et al., 2010; Ozaki et al., 2011; Yasuda et al., 2014; Clarkson et al., 2017b), and improved by significantly more than the comparison group in three of these studies (Abe et al., 2010; Yasuda et al., 2014; Clarkson et al., 2017b). This may be expected due to the known ability of BFR exercise to enhance muscle strength and the relative contribution of strength in this measure (McCarthy et al., 2004; Slysz et al., 2016). Similarly, performance on the TUG, a measure of dynamic balance and mobility (Bohannon, 2006), improved following BFR exercise interventions in seven of the eight included studies (by between 10 and 16%) (Yokokawa et al., 2008; Abe et al., 2010; Ozaki et al., 2011; Araujo et al., 2015; Bryk et al., 2016; Clarkson et al., 2017b; Ferraz et al., 2018; Jorgensen et al., 2018), and improved by significantly more than the comparison group in five of these studies (Yokokawa et al., 2008; Abe et al., 2010; Ozaki et al., 2011; Araujo et al., 2015; Clarkson et al., 2017b). Performance on the TUG is known to be inhibited by reduced pelvic, lower limb and core muscle strength (Binda et al., 2003). Therefore, though BFR training primarily affects tissues distal to the restrictive cuff, the common improvement in TUG between studies may suggest that the interventions employed may also enhance the strength of synergist and stabilizer muscles such as pelvic and core musculature (Slysz et al., 2016; Lixandrao et al., 2018). As these measures provide the most insight into the efficacy of BFR exercise for improving objective measures of physical function indicative of ADL among the available literature, there is support for both BFR-RT and BFR-AT for improving physical function. This is especially true given that the reduced intensity and physiological stress of the exercise is suitable for populations that are most in need of improvements in physiological function.

### Limitations of the Included Studies

**Figure 2:**
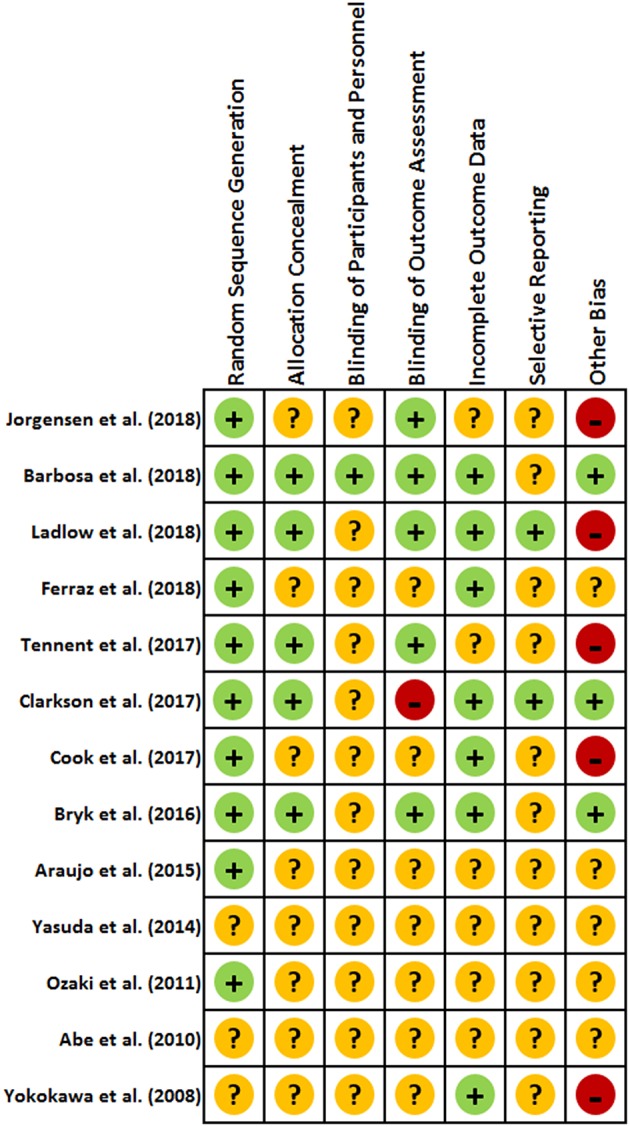
Risk of bias assessment for included studies evaluating changes in objective measures of physical function following exercise intervention combined with blood flow restriction.

While this review included good quality randomized controlled trials, a moderate risk of bias in some studies was still present (Figure 2). Despite an indication of randomization, three studies inadequately reported the methodology with which participants were randomized (Yokokawa et al., 2008; Abe et al., 2010; Yasuda et al., 2014), and only five studies specified the method of allocation concealment (Bryk et al., 2016; Clarkson et al., 2017b; Tennent et al., 2017; Barbosa et al., 2018; Ladlow et al., 2018). Perhaps most notably, blinding of participants and study personnel was of concern (Schulz et al., 1995). However, blinding of participants is something that may be difficult to account for with training studies, particularly when the comparison group is either a different format of exercise, or an inactive control and is inherent in many training studies. Additionally, only three studies indicated the use of the intention-to-treat principle (Barbosa et al., 2018; Ferraz et al., 2018; Jorgensen et al., 2018); only seven studies included sample size calculations (Araujo et al., 2015; Bryk et al., 2016; Cook et al., 2017; Barbosa et al., 2018; Ferraz et al., 2018; Jorgensen et al., 2018; Ladlow et al., 2018); and only eight sufficiently reported compliance (Yokokawa et al., 2008; Bryk et al., 2016; Clarkson et al., 2017b; Cook et al., 2017; Barbosa et al., 2018; Ferraz et al., 2018; Jorgensen et al., 2018; Ladlow et al., 2018). Two studies presented only the mean and variance of the change in measures of physical function from before to after the intervention, but did not report the means and variance for both the pre- and post-intervention time points (Cook et al., 2017; Ladlow et al., 2018). This potentially limits the understanding of how impactful the demonstrated change scores were, and may limit the interpretation of how low the level of physical function must be in order to garner an advantage from this training modality.

From a broader perspective, the overall low number of studies and lack of homogeneity among them makes it difficult to collectively analyse and interpret the results for many of the less commonly used measures of physical function. However, the commonly used measures in the 30STS and TUG provide substantial evidence supporting the efficacy of BFR exercise for improving physical function, particularly functional strength, dynamic balance and mobility. This may indicate that future studies need to assess the influence of BFR training interventions on performance of a more holistic battery of measures of physical function. The lack of homogeneity among included studies is also the primary reason a meta-analysis was not attempted in addition to the present review. Among the thirteen included studies, there were seven different populations examined. While exercise may generally have a similar effect, there are likely physiological differences between these different populations that are extraneous variables that make it difficult to provide generalizations about BFR exercise for any single population. This is the primary reason for why reporting minimal clinically important difference (MCID) is difficult for the outcomes in this review, as this is something that can be markedly variable depending on the population in question, and to try to imply a blanket MCID across multiple populations may be misleading. This would be a valuable addition to the reporting of the outcomes for objectively measured physical function, and future research should provide an indication of what is considered a MCID for the populations they assess. Likewise, large variability in the exercise prescriptions (exercise training modalities, pressure applications and restriction durations) used among the included studies makes it difficult to elucidate whether any single prescription is particularly useful or more efficacious than another. However, it is difficult to recommend specific exercise prescriptions as this is variable in response to the population group being examined, and the training objectives of note. Perhaps a more apt recommendation is for more repetition of similar exercise prescriptions for specific training objectives among single populations as a means of enhancing evidence that is touched on by previous findings. Finally, while the 30STS and TUG were more frequently examined, making it easier to draw conclusions about the general effects of BFR exercise and performance on these measures, no other single measure of physical function identified in this review was assessed in more than two studies. Thus, more studies examining similar key measures of physical function are required when assessing outcomes from BFR training.

## Conclusions

Physical function is generally an undervalued and infrequently measured outcome among blood flow restriction exercise studies, which traditionally focus on muscle mass and strength outcomes. Task-specific measures of physical function indicative of activities of daily living may be more clinically relevant and applicable for evaluating the management or progression among chronic disease and other clinical populations (Groll et al., 2005). The results of this review indicate that blood flow restriction exercise has potential for improving physical function measured by tasks reflective of everyday activities. However, the inconsistency regarding target populations, exercise prescription, and outcome measures assessed demonstrates a need for greater research focus and consistency, particularly within specific populations in future research. Regardless, blood flow restriction exercise is frequently purported to be of significant benefit for chronic disease and other clinical populations due to reduced mechanical, haemodynamic and perceptual stress. In addition to these reduced physiological stresses, the present review suggests that blood flow restriction exercise may provide equivalent, or even greater stimulus for improving physical function than some non-blood flow restriction equivalent or more traditional exercise prescriptions. Therefore, this review supports the utilization of blood flow restriction exercise in clinical rehabilitation or the management of chronic diseases, particularly with regard to improving measures of physical function indicative of everyday tasks that utilize strength, dynamic balance and mobility.

## Author Contributions

MC was primarily responsible for the conception of and rationale for the review. MC, AM, and SW contributed to the search strategy, the selection criteria, the review, and writing of the manuscript. MC and AM completed the database searches, study selection, and assessment of bias. MC completed data extraction and compilation of results.

### Conflict of Interest Statement

The authors declare that the research was conducted in the absence of any commercial or financial relationships that could be construed as a potential conflict of interest.
