# Supplementary material for: Chronic Blood Flow Restriction Exercise Improves Objective Physical Function: A Systematic Review
Source: Front Physiol. 2019 Aug 21;10:1058. doi: 10.3389/fphys.2019.01058 (PMC6712096; doi:10.3389/fphys.2019.01058)
Supplement: Supplementary file 1 [file Table_1.DOCX]

**Databases:**

EMBASE, Springer, Medline, CINAHL, SPORT Discus

**Filters:** Human, English, randomised controlled trial, Controlled trial, Clinical trial, Controlled clinical trial, Journal/Journal article/Academic journal

***Search strings edited to format required for database. Same search terms, possibly different wildcards, operator layout, or stemming requirements***

**Search String (CINAHL):**

((“physical function” OR “function*” OR "strength gain*" OR "strength*") AND (“exercis*” OR “aerobic” OR “endurance*" OR "walk*" OR "run*" OR "cycl*" OR “resistance train*” OR "resistance*" OR "resist*" OR “strength train*” OR “strength*” OR “weight train*” OR “circuit train*” OR “weight lift*” OR "train*") AND ("muscle*" OR "musc*" OR "myo*") AND (“blood restrict*” OR “blood flow restrict*” OR “occlusion*” OR “occlud*” OR “restrict blood*” OR “arterial restrict*” OR “venous restrict*” OR “cuff*” OR “hypoxi*”) NOT “cardiac” NOT “cardi*” NOT "mouse" NOT "rat*" NOT "animal*" NOT "horse*" NOT "mice") OR ("kaatsu*")

***Manual Filter: English, Academic Journals***

**Search String (SPORT Discus):**

((“physical function” OR “function*” OR "strength gain*" OR "strength*") AND (“exercis*” OR “aerobic” OR “endurance*" OR "walk*" OR "run*" OR "cycl*" OR “resistance train*” OR "resistance*" OR "resist*" OR “strength train*” OR “strength*” OR “weight train*” OR “circuit train*” OR “weight lift*” OR "train*") AND ("muscle*" OR "musc*" OR "myo*") AND (“blood restrict*” OR “blood flow restrict*” OR “occlusion*” OR “occlud*” OR “restrict blood*” OR “arterial restrict*” OR “venous restrict*” OR “cuff*” OR “hypoxi*”) NOT “cardiac” NOT “cardi*” NOT "mouse" NOT "rat*" NOT "animal*" NOT "horse*" NOT "mice") OR ("kaatsu*")

***Manual Filter: English, Academic Journals***

**Search String (Medline):**

((“physical function” OR “function*” OR "strength gain*" OR "strength*") AND (“exercis*” OR “aerobic” OR “endurance*" OR "walk*" OR "run*" OR "cycl*" OR “resistance train*” OR "resistance*" OR "resist*" OR “strength train*” OR “strength*” OR “weight train*” OR “circuit train*” OR “weight lift*” OR "train*") AND ("muscle*" OR "musc*" OR "myo*") AND (“blood restrict*” OR “blood flow restrict*” OR “occlusion*” OR “occlud*” OR “restrict blood*” OR “arterial restrict*” OR “venous restrict*” OR “cuff*” OR “hypoxi*”) NOT “cardiac” NOT “cardi*” NOT "mouse" NOT "rat*" NOT "animal*" NOT "horse*" NOT "mice") OR ("kaatsu*")

***Manual Filter: Clinical Trial, Controlled clinical trial, randomised controlled trial***

**Search String (Springer):**

(“physical function” OR function OR “strength gain” OR strength) AND (exercise OR aerobic OR endurance OR walking OR running OR cycling OR “resistance train*” OR resistance OR “strength train*” OR strength OR “weight train*” OR “circuit train*” OR “weight lift” OR train) AND (muscle OR myo*) AND (“blood restrict*” OR “blood flow restrict*” OR occlusion OR occlude OR “restrict blood” OR “arterial restrict*” OR “venous restrict*” OR “cuff” OR hypoxi*) NOT cardi* NOT cardiac NOT mouse NOT rat NOT animal NOT horse NOT mice OR kaatsu

***Manual Filter:*** *English, Article*

**Search String (EMBASE):**

((“physical function” OR “function*” OR "strength gain*" OR "strength*") AND (“exercis*” OR “aerobic” OR “endurance*" OR "walk*" OR "run*" OR "cycl*" OR “resistance train*” OR "resistance*" OR "resist*" OR “strength train*” OR “strength*” OR “weight train*” OR “circuit train*” OR “weight lift*” OR "train*") AND ("muscle*" OR "musc*" OR "myo*") AND (“blood restrict*” OR “blood flow restrict*” OR “occlusion*” OR “occlud*” OR “restrict blood*” OR “arterial restrict*” OR “venous restrict*” OR “cuff*” OR “hypoxi*”) NOT “cardiac” NOT “cardi*” NOT "mouse" NOT "rat*" NOT "animal*" NOT "horse*" NOT "mice") OR ("kaatsu*")

**For Filters:** …AND ('clinical trial'/de OR 'controlled clinical trial'/de OR 'controlled study'/de OR 'human'/de OR 'major clinical study'/de OR 'normal human'/de OR 'randomized controlled trial'/de OR 'randomized controlled trial (topic)'/de) AND 'article'/it
